# Assessment and Treatment of Varus Foot Deformity in Children with Cerebral Palsy: A Review

**DOI:** 10.3390/jcm15031147

**Published:** 2026-02-02

**Authors:** Robert M. Kay, Susan A. Rethlefsen

**Affiliations:** 1 Jackie and Gene Autry Orthopedic Center, Children’s Hospital Los Angeles, Los Angeles, CA 90027, USA; rkay@chla.usc.edu; 2 Keck School of Medicine, University of Southern California, Los Angeles, CA 90033, USA

**Keywords:** cerebral palsy, varus foot deformity, assessment, treatment, surgery

## Abstract

Cerebral palsy (CP) is a developmental disability caused by injury to the fetal or infant brain, affecting between 1.6 to 3.7 per 1000 live births worldwide. Ambulatory patients with cerebral palsy experience various gait problems, for which they seek treatment from medical professionals. Varus foot deformities are among the most problematic for patients. Varus foot deformity is characterized by the inner border of the foot being tilted upward and the hindfoot inward, increasing weightbearing on the lateral aspect of the foot. This positioning increases weight-bearing pressure under the lateral (outside) of the foot and often under the fifth metatarsal head when walking. As such, varus foot deformity can contribute to in-toeing, make shoe and brace-wearing difficult and painful, compromise gait stability, and sometimes lead to metatarsal fractures. Current knowledge of CP etiology and classifications, as well as principles and advances in assessment and treatment decision making for varus foot deformities, are outlined in this narrative review. In younger children with flexible deformities, non-operative interventions such as bracing, botulinum toxin injection, and serial casting are effective. The literature and expert consensus suggest that, if possible, surgery should be delayed until after the age of 8 years. When surgery is indicated, soft tissue procedures are used for flexible deformities. In addition to the soft tissue procedures, bone surgery is needed for rigid deformities. Careful pre-operative foot assessment is needed, including assessment of deformity flexibility and range of motion, X-rays, and computerized gait analysis if possible. Strategies are presented for thorough assessment when gait analysis is not available or feasible. Research reports of surgical outcomes for soft tissue and bony correction are positive, but should be interpreted with caution. The quality of evidence on surgical outcomes is compromised by use of varying research design methods and selection of outcome measures, with few including measures of function or patient-reported outcomes. It is recommended that surgical outcome be assessed using standardized assessment tools, such as the Foot Posture Index, which have had their validity and reliability established. Recent advances in 3D kinematic foot model development and musculoskeletal modeling have the potential to greatly improve surgical outcomes for patients with CP.

## 1. Introduction

Cerebral palsy (CP) describes a group of disorders of the development of movement and posture, often including various comorbidities involving speech, cognition, and behavior, caused by injury to the fetal or infant brain. Brain lesions involved in CP are not progressive, but their manifestations do change with growth and maturation [1]. The prevalence of CP has been estimated at 1.6 per 1000 live births in high-income regions and 2.3–3.7 per 1000 live births in low- to middle-income regions of the world [2]. Though CP is primarily caused by factors such as prematurity and low birth weight, recent research has revealed that genetic factors are implicated in 31% of cases [3].

Cerebral palsy is classified based on the type of motor impairment and limb distribution, as well as gross motor functional ability. The most common motor impairment is spasticity. Distribution of limb involvement in patients with spastic CP is classified as diplegia (primarily bilateral lower extremities) in 13–25%, hemiplegia (upper and lower limbs, one side) in 21–40%, and quadriplegia (all limbs, or total body) in 20–43%. Less common motor impairment types include dyskinesia (athetosis, dystonia) in 14% of patients, and ataxia in 4–13% [4]. There can be mixed patterns of motor impairment as well.

The most commonly used functional classification for children with CP is the Gross Motor Function Classification System (GMFCS), which groups patients based on gross motor functional performance and age. There are five levels, ranging from independent ambulation on all terrains (GMFCS level I) to non-ambulatory, with no head or neck control, and full dependence on others for mobility (GMFCS level V). The GMFCS is applicable to children with CP from 0–18 years of age, and accounts for changes in preferred mobility in the teenage years due to changes in activity preferences, physical and environmental situations, and personal choices [5]. Risk of hip subluxation and severe scoliosis are directly correlated with GMFCS levels, with high rates seen in patients at GMFCS levels IV and V and very low rates in those at GMFCS levels I and II [6,7]. There is also evidence of gross motor functional decline among children functioning at GMFCS levels III, IV, and V, likely due to physical factors such as changes in the body-weight-to-muscle-strength relationship as children grow and mature. More ambulatory children (GMFCS levels I and II) can be expected to maintain the gross motor skills attained during childhood into adulthood [8]. Studies employing 3D computerized gait analysis (3D-CGA) confirm that the number and severity of gait deviations and gait problems increase with greater degrees of functional limitation in CP (higher GMFCS level) [9,10].

**Figure 1:**
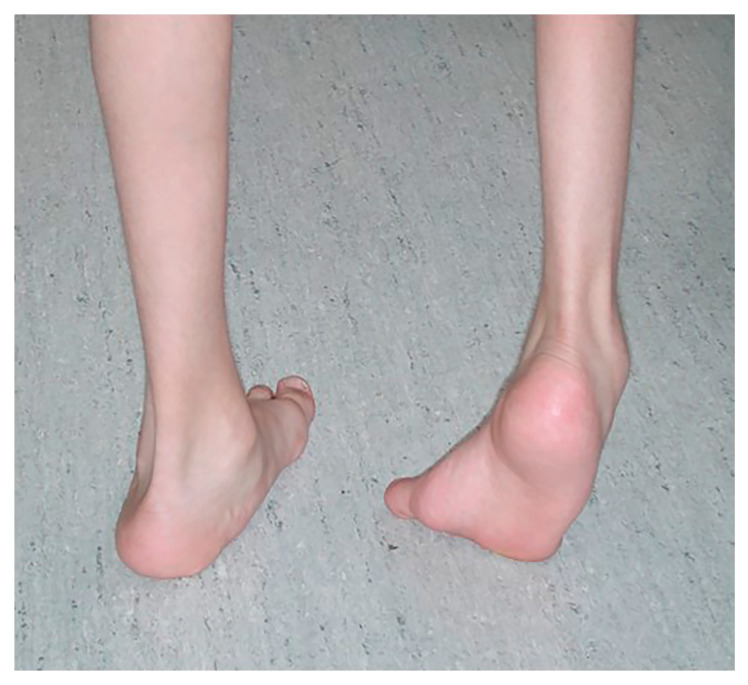
Varus foot, characterized by the inner border of the foot being tilted upward and the hindfoot inward and increased weightbearing on the lateral aspect of the foot. Equinus (excessive plantarflexion) is also often present. Timing of the varus foot motion is assessed during gait (swing versus stance phase, or continuous) by observing the patient from the front, back, and sides in stance and swing phases. (See Section 3, Figure 2).

**Figure 2:**
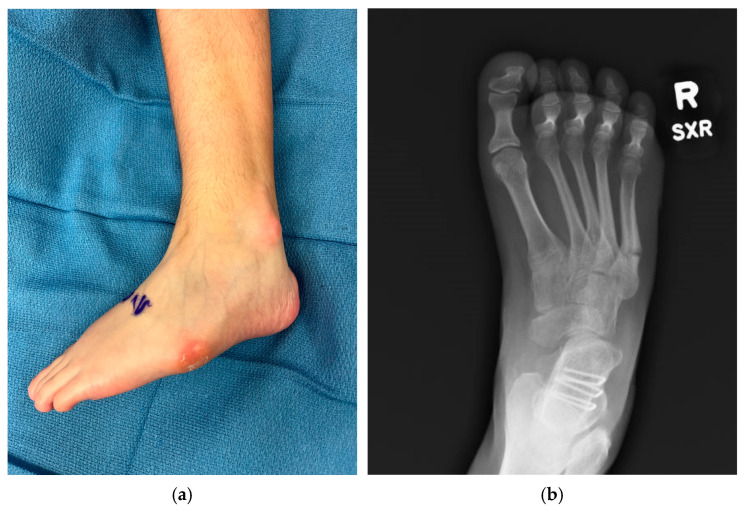
Examples of negative sequelae of varus foot deformities. (**a**) Varus foot with evidence of skin breakdown at base of 5th metatarsal and lateral malleolus from poor bracing tolerance, 13-year-old female; (**b**) 5th metatarsal base fracture in a 17-year-old female.

Foot and lower leg pain are common in patients with cerebral palsy (CP), particularly those who are ambulatory [11], and foot deformities may be one factor contributing to this pain. Though varus foot deformities are less common than valgus deformities in these patients, they can lead to significant problems with tolerating shoe and brace wear, especially as children grow and their deformities become stiffer. Varus foot deformity is characterized by the inner border of the foot being tilted upward and the hindfoot inward, with increased weightbearing on the lateral aspect of the foot (Figure 1). Varus foot is often accompanied by equinus (excessive plantarflexion). Excessive weight-bearing pressure under the lateral aspect of the foot and the fifth metatarsal head when walking leads to pain and occasionally to skin breakdown from pressure from an orthotic, as well as metatarsal fractures (Figure 2). Though both varus and valgus foot deformities can result in pain, as well as problems with shoe and/or brace wear, varus feet are often more problematic for children with CP because they also typically interfere with stability in stance, one of the five prerequisites of normal gait [12]. Varus foot deformity has been reported in 6% of children with CP in the Swedish CPUP registry [13]. The prevalence of varus foot has been reported to be approximately 10% of all children presenting to a gait lab in a tertiary medical center [9], and is more common in children with in-toeing who have hemiplegia (43%) than in those with diplegia or quadriplegia (8%) [14]. Though more common in children with hemiplegia, varus foot deformity can also occur unilaterally or bilaterally in children with diplegia or quadriplegia. Varus foot may take one of three main forms: 1. The foot is in a neutral alignment at rest, but goes into varus alignment with muscle activity (also called “dynamic varus”). 2. The foot rests in varus alignment, which is often exacerbated during activity (also called “flexible structural varus”). 3. The foot sits in a varus position which cannot be corrected with manipulation (also called “rigid varus.”) The deformity often starts as “dynamic”, progresses to a “flexible structural” deformity, and ultimately becomes rigid over time.

Regardless of the child’s type of CP (hemiplegia or di- or quadriplegia) or the flexibility of the varus foot, the varus foot deformity stems from muscle imbalance with overpull of the invertors (anterior and/or posterior tibialis) relative to the evertors (peroneals). The problem of muscle imbalance in children with CP is that it typically leads to the development of musculoskeletal deformities over time throughout the child’s body, not just at the levels of the foot and ankle. It should be noted that studies on the prevalence of various gait problems in patients with CP have often been performed in high-income countries with greater access to medical care. As such, the prevalence of varus foot may be different low- and middle-income countries. Varus foot deformity negatively impacts gait. It contributes to in-toeing, often compromises stability in stance, and is problematic for children as well as adults with CP, who often have baseline challenges in balance, strength, and motor planning.

Prior to the advent of 3D CGA, children with CP often underwent orthopedic surgery at a single level or joint at a time, repeatedly throughout their childhood (a phenomenon known as Birthday Syndrome). Assessment and treatment of gait disorders have dramatically improved, with increased use of 3D CGA for pre-operative planning for children with CP being considered for orthopedic surgery. 3D CGA enables dynamic assessment of motion occurring at multiple joints and in multiple planes of motion simultaneously, allowing surgical intervention at multiple levels simultaneously (single event multi-level surgery, or SEMLS). 3D CGA is more accurate than visual assessment or other static measures of ambulation [15,16,17]. 3D CGA improves clinicians’ ability to assess transverse plane gait problems at the level of the hip and pelvis [18,19]. Surgical outcomes are improved when recommendations based on 3D CGA are followed [20]. A systematic review of articles on 3D CGA demonstrated its efficacy for altering surgical plans and increasing surgeon confidence in their treatment plans. Adherence to surgical plans based on 3D CGA has been shown to improve surgical outcomes [21]. Dynamic electromyography data from the anterior and posterior tibialis muscles during 3D CGA is crucial. It is needed to determine the muscular cause of varus foot positioning and to optimize surgical outcomes [22].

The purposes of this narrative review are to outline the current methods of identification and assessment of varus feet in patients with CP, current treatments and their outcomes, and directions for future research on the treatment of these complex and problematic foot deformities.

## 2. Etiology of Varus Feet

Muscle balance at the levels of the foot and ankle is compromised in children with CP, as it is at other levels in the upper and lower extremities and the trunk. Varus foot deformities in these children typically develop over time. In a longitudinal study, Church et al. reported on plantar pressure data from children with CP and found that most developed normal or valgus foot deformities as they aged. However, 6–8% of patients at GMFCS levels I/II (independently ambulatory) and 25% of children functioning at GMFCS level III (aided ambulators) developed varus foot deformities over an approximately 18-year period of time [23]. However, it should be noted that this study did not control for the impact of treatments such as botulinum toxin injections, serial casting, bracing, or surgery. Results may be different in other centers or in an untreated population.

The traditional teaching was that posterior tibialis muscle spasticity or overactivity is the cause of varus deformity in 90% of cases. However, dynamic electromyography during gait, including fine wire electromyography of the anterior and posterior tibialis, has demonstrated that muscle contributions to the varus foot include the anterior tibialis in two thirds of cases (one third of cases is isolated anterior tibialis and one third is combined anterior and posterior tibialis), while the posterior tibialis is the isolated contributor in only one third of cases (Table 1) [22,24]. Very mild cases of varus foot can be due to overpull/contracture of the triceps surae and can resolve with lengthening of the gastrocnemius or Achilles tendon [25].

## 3. Assessment

The foot deformity should be evaluated in the context of the child’s overall gait, alignment, and function. In children with CP, problems are rarely only present at one level. In-toeing is a common gait deviation recognized by patients, parents, and clinicians, and in-toeing is known to have multiple causes in addition to varus foot posture [14]. Therefore, it is paramount to evaluate all levels (pelvis, hip, knee, and ankle) and all planes of motion when evaluating a child and their gait.

A recent consensus study involving 16 experienced pediatric orthopedic surgeons with expertise in assessment of patients with CP yielded guidelines for evaluation of varus foot deformities [26]. Physical examination should include assessment of the flexibility of the deformity. Whereas for children with typical balance and coordination, the Coleman block test (having them stand on a block with the medial column off the block) can be used, that is not a reasonable option in children with CP. Instead, the simplest and safest way to assess the flexibility of the deformity is via manual manipulation. The hindfoot can be grasped and everted to assess for hindfoot flexibility. If the talar head is very prominent laterally, the foot can be manipulated to assess whether the talus can be realigned more appropriately with the navicular. The confusion test can be helpful to determine whether the anterior tibialis is contributing to the varus foot. The child is asked to flex their hip actively in a seated position. Davids et al. confirmed that the anterior tibialis will fire as part of a mass action pattern as the child flexes their hip, though firing of the muscle is not, of itself, pathologic [27]. However, if the confusion test results in significant varus positioning of the foot, then the anterior tibialis is likely a significant contributor to varus during gait.

**Figure 3:**
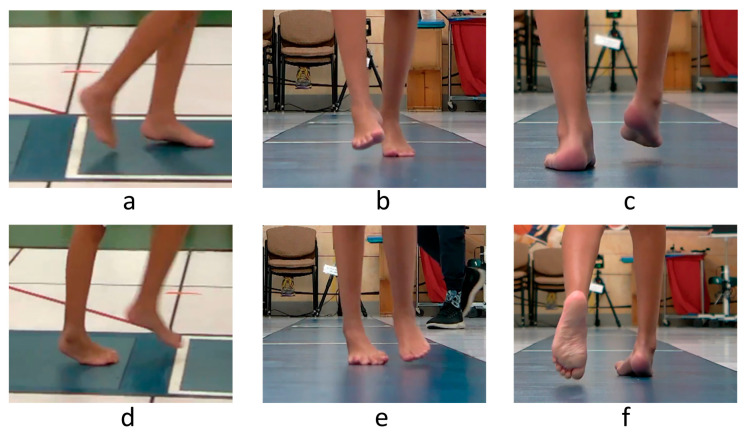
Observational assessment of right foot position during gait of a sample patient. Swing phase is shown in the top row and stance in the bottom row of images. The right foot exhibits excessive plantarflexion (**a**,**d**) and forefoot (**b**,**e**) and hindfoot (**c**,**f**) varus in the swing and stance phases of gait.

Observational gait assessment can be used to assess the timing of the varus foot motion during gait (swing versus stance phase, or continuous) and also the severity of the deformity. Plantarflexion and forefoot and hindfoot varus should be assessed from the front, back, and sides in stance and swing phases (Figure 3). Standardized observational gait assessment tools such as the Edinburgh Visual Gait Scale (EVGS) can be used either in the clinic or for videos for this purpose [28]. The EVGS demonstrates good inter-rater (60–90%) and intra-rater (64–92%) reliability [15].

For children who are candidates for surgery, the combination of plain radiographs and 3D CGA is the gold standard for assessment. The consensus is that radiographs should be performed on weightbearing whenever possible (GMFCS levels I-III), and either assisted or simulated weightbearing if not possible (GMFCS IV-V). AP and lateral views of the foot and AP views of the ankle have been recommended [26]. Children with hemiplegia may have underlying limb length discrepancy, which should also be investigated clinically or radiographically. A compensatory elevated ipsilateral hemipelvis (an accommodation for equinovarus) often creates the appearance of a limb length difference when no structural difference is present.

Standing radiographic measures do not correlate well with dynamic measures of foot and ankle position during gait in pathologic conditions [29]. Therefore, 3D CGA is also highly recommended. Physical examination performed in a gait analysis laboratory includes a full examination of range of motion, muscle strength and selective control, and muscle spasticity. It also often includes assessment of standing foot position using standardized tools such as the Foot Posture Index-6 (FPI-6). The FPI-6 is useful for quantifying the severity of deformity as well as the contributing components of the deformity. The FPI-6 has had its validity established, as well as excellent inter- and intra-rater reliability in children and adults [30,31,32,33]. 3D joint motion (kinematics) and force data (kinetics) provide specific information about the amount and direction of motion at all joints simultaneously. Dynamic electromyography provides information about the timing and intensity of muscle contraction during gait. Sampling of the anterior and posterior tibialis muscles is necessary to determine their contributions to varus foot motion during gait, allowing targeting of intervention to the appropriate source (anterior and/or posterior tibialis) and potentially improving surgical outcomes. Fine wire electromyography can be uncomfortable and cause anxiety for some patients, as the procedure is invasive. However, research has demonstrated that the presence of fine wire electrodes does not alter temporal and spatial parameters or foot/ankle motion during gait as measured using a multi-segment foot model in children with hemiplegic CP [34]. Pre- and post-operative pedobarography (foot plantar pressure) is useful for documenting surgical outcomes.

Standard 3D CGA involves a simplified model of the foot as a rigid segment, providing no information about the position or motion of joints within the foot itself (subtalar, mid-tarsal, and tarso-metatarsal). However, in recent years, multi-segment foot models have been developed, validated, and refined, proving useful in further elucidating foot deformities [35,36,37]. These models examine the 3D motion of the hindfoot, forefoot, and hallux, and some include mid-foot motion. Using the Amsterdam model, Schallig et al. determined that patients with CP and varus feet had more ankle varus and Chopart’s joint inversion and adduction than typically developing peers, with little difference at the Lisfranc joint [35]. Krzak et al. identified five subtypes of varus feet in patients with hemiplegic CP using multisegment foot modeling. These subtypes had varying combinations of hindfoot and forefoot alignment and movements in all three planes of motion [38]. Such patient-specific information about various components of foot motion may allow better treatment decision making and better surgical outcomes. However, sophisticated multi-segment foot models are not currently in wide use and are not always feasible (severe deformities or very small feet can obscure marker visibility). A simpler two-segment foot model using only one additional motion capture marker and measuring forefoot and hindfoot motion is currently being validated, which may be more useful for smaller feet or more severe deformities [39,40]. Since development of multi-segment foot models is relatively new, their validity for use in clinical decision making and their impact on surgical outcomes have not yet been reported. Several models exist, and there is no consensus on which is superior. More research is needed on the clinical impact of multi-segment foot models.

Unfortunately, computerized gait analysis is not widely available. 3D CGA requires specially trained staff, including clinicians able to perform dynamic electromyography testing. There are currently only 15 laboratories worldwide that are accredited by the Commission for Motion Laboratory Accreditation and use standardized procedures for their testing. The typical cost of 3D CGA is approximately $3500 [41], and is often not covered by insurance. When 3D CGA is not feasible, careful examination as outlined above, videotaping the patient from the front and sides for later review, plus use of the FPI-6 and EVGS. are recommended.

## 4. Treatment

The varus deformity almost invariably starts as a flexible deformity; with time, the deformity often becomes fixed, and secondary bone changes develop. In younger children, and in those with flexible deformities, non-invasive treatments are used. When non-invasive treatments are no longer effective, soft tissue surgery may be warranted, with or without concomitant bone procedures, depending on deformity severity.

### 4.1. Non-Operative Treatment

**Figure 4:**
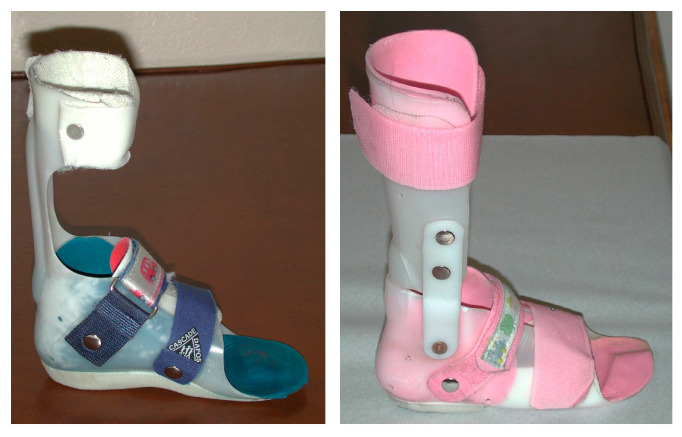
Examples of ankle foot orthoses with wrap-around foot components ((**left**)—posterior leaf spring, (**right**)—hinged). The wrap-around foot component provides complete contact with the foot for better control of varus position and to minimize point-pressure within the brace.

Non-operative care is typically the first line of treatment for children with varus foot deformities. For dynamic varus (without fixed soft tissue contracture or bone deformities), appropriate bracing is the main course of treatment. Commonly, this requires a brace with a wrap-around hindfoot component to capture the varus foot (Figure 4). The typical indications for brace use include one or both of the following: 1. Brace use is anticipated to enhance gait and function, and/or 2. brace use is expected to minimize the risk of progressive deformity. Wrap-around foot components provide complete contact with the foot to better control varus foot position and to minimize point-pressure within the brace. Sagittal plane control depends on the child’s tendency toward equinus. If the varus deformity is very mild and limited to the coronal plane, a supra-malleolar orthosis may be sufficient. If there is a strong tendency for plantarflexion and associated knee hyperextension to occur in the stance, an orthotic that strongly resists plantarflexion (such as a solid ankle foot orthosis, or AFO) is needed. For milder cases of supple equinovarus, some flexibility in the sagittal plane can be allowed, such as a posterior leaf spring or even a hinged AFO.

Other treatments which may be helpful are the use of botulinum toxin injections with or without serial casting, and physical therapy. Botulinum toxin injections are effective in decreasing spasticity and may aid in patient tolerance of bracing or serial casting. However, they are not effective in treating fixed contractures [42]. Botulinum toxin injections may be used in an attempt to control dynamic deformities and delay or possibly avoid surgery. In their early stages, fixed contractures can be managed by serial casting and physical therapy. However, once non-operative interventions are insufficient and bracing cannot be tolerated, surgery may be indicated.

### 4.2. Operative Treatment

Ideally, surgical intervention should be delayed until a child reaches 7–10 years of age, when they have likely achieved their peak level of gross motor skills, and when non-surgical options are no longer effective. The author’s preference is to wait until the child has reached a plateau in gross motor skill development of at least 6 months to be certain that the musculoskeletal problems present are interfering with further progress.

Prior to opting for surgery, parents and families should be fully educated on the likelihood of the increased burden on them to care for their child post-operatively, and the need for increased therapy services after surgery. While there is no consensus on the optimal post-operative rehabilitation regimen for children with CP [43], it is likely to be intensive, as recovery is improved with increased physical therapy treatment [44]. SEMLS is the standard of care for children with varus foot deformities, as these are rarely isolated problems. Full gross motor functional change after SEMLS with or without varus foot deformity correction may not be achieved until 2 years post-operatively [45]. Knowledge of this information is important to help families prepare for recovery and manage expectations for their child’s surgical outcome.

In cases where equinus contractures are present, triceps surae lengthening should be performed. If contracture is present with the knee extended (gastrocnemius) but not when flexed (soleus), gastrocnemius recession is indicated. If fixed contracture is present both with the knee flexed and extended, tendo-Achilles lengthening is needed. Very mild cases of varus foot (such as in younger children) can be caused by overpull/contracture of the triceps surae, and can often be corrected with lengthening of the gastrocnemius or Achilles tendon alone [25]. However, aggressive triceps surae lengthening should be avoided, especially in patients with bilateral involvement. The natural history of gait progression in bilateral CP is for ankle dorsiflexion and knee flexion to increase with increased age and body weight [9,46,47,48,49]. Aggressive triceps surae lengthening may exacerbate this problem.

Soft tissue balancing for varus feet is typically necessary regardless of whether the deformity is flexible or rigid. The soft tissue balancing includes addressing problems with the anterior tibialis, posterior tibialis, or both. As outlined above, 3D CGA including dynamic EMG facilitates this decision-making process. If 3D CGA data are not available, then the confusion test noted above can help determine whether the anterior tibialis is a contributor to the deformity. In some centers, botulinum toxin injection into the posterior tibialis is used pre-operatively in an attempt to simulate potential outcomes of posterior tibial tendon lengthening in order to enhance pre-operative planning.

For posterior tibial tendon surgery, good results have been reported following either posterior tibial tendon lengthening (PTTL) or split posterior tibial tendon transfer (SPOTT). There is no good data to support the superiority of one technique over the other. A recent literature review cited risk factors for failure after SPOTT included age <8 years at surgery, incorrect tensioning of the tendon transfer, residual spasticity, and untreated bone deformity [50]. Based on our experience, we typically perform PTTL more commonly than SPOTT. SPOTT is typically used for more severe deformities in younger children (usually <10 years old) and PTTL for the majority of other children needing posterior tibial tendon surgery.

Posterior tibial tendon lengthening (PTTL) is typically performed with fractional lengthening of the posterior tibialis (via intramuscular tenotomy) a few centimeters proximal to the medial malleolus [51].

Anterior tibial tendon surgery is performed via split transfer (SPLATT). Lengthening of the anterior tibialis is not an option, as that would result in (and/or exacerbate) equinus. Split transfer may be to bone or soft tissue. The authors prefer transfer to the peroneus tertius (which is present in approximately 90% of children) as this transfer functions well, tension can be optimized, and it avoids complications associated with transfer to bone such as pressure sores or hardware failure and avoids the use of expensive implants [51]. In patients in whom the peroneus tertius is not present or of sufficient size, the peroneus brevis is an excellent alternative, as the dorsal half can be used to receive the SPLATT. Either the peroneus tertius or brevis is an excellent recipient tendon, and previous work has shown maintained or improved dorsiflexion strength in 77% of 133 patients with CP who underwent SPLATT to the peroneus tertius or brevis. Overcorrection into valgus is uncommon (8.7%) with this procedure [52].

In general, the combination of posterior tibial tendon lengthening (PTTL) and split anterior tibial tendon transfer (SPLATT) is a good choice of procedure for soft tissue balancing in children with CP and varus foot deformity when 3D CGA is not available. This combination has a high rate of good correction with a small risk of overcorrection.

Despite overall positive surgical outcomes, recurrence and overcorrection can occur. A systematic review of studies reporting on outcome of split anterior and posterior tibialis tendon transfers using 3D CGA revealed rates of varus recurrence of 12%, overcorrection into valgus of 2.8%, and need for revision surgery of 11.8% [53]. Reviewed as a whole, studies on the outcome of tendon transfers in the literature are limited by inconsistent definitions of recurrence, failure, and successful outcomes, and failure to include a standardized measure of function as well as patient-reported outcome measures [50,53,54].

For rigid deformities, osseous surgery is needed in combination with appropriate soft tissue balancing. In general, calcaneal osteotomy is the workhorse for bone correction. This can be accomplished via a Dwyer type lateral closing wedge osteotomy of the calcaneus or via a lateral calcaneal slide, with both types allowing for good correction [55,56]. We more commonly advocate for lateral slide osteotomies as they are technically easier and faster and can be fixed with smooth Kirschner wires which are easily removed in the office at the 3–4-week post-operative visit. Dwyer osteotomies may be limited in the size of the lateral wedge that can be removed, require a wider exposure of the lateral wall of the calcaneus, and typically rely on more expensive fixation, which typically remains in the child unless a second surgery is later performed for hardware removal. For some severe deformities, a Dwyer closing wedge osteotomy may be performed at the level of the lateral slide.

Triple arthrodesis may occasionally be indicated in children with CP and severe, rigid deformity who function at GMFCS levels IV and V. It may also be considered as a salvage procedure in severe, recurrent deformities in children functioning at GMFCS level III. Trehan et al. reported satisfaction in 95% of patients with CP (primarily functioning at GMFCS I and II) at a mean of 22.1 years following triple arthrodesis [57]. Triple arthrodesis is typically avoided in higher functioning patients due to the risk of arthritic changes in the ankle and other joints of the foot and ankle during their lifetimes, and Trehan et al. reported radiographic evidence of ankle arthritis and midfoot arthritis in 11.8% and 3.5% (respectively) of their cohort at a mean final radiographic follow-up of 21.6 years [57].

## 5. Conclusions

Varus foot deformities in children with CP are less common than valgus deformities. Nevertheless, they can be a significant cause of pain and gait dysfunction in patients who have them. Much work has been done over the past decade to document the development of foot deformities, including varus deformity, as children age, as well as to identify the muscular contributors to dynamic varus during gait. This knowledge has led to improved treatment outcomes.

All children with CP require ongoing follow-up with an orthopedic surgeon familiar with the care of children with CP. In addition, significant progressive foot deformity and/or brace wear difficulties in these children should prompt the pediatrician, physiatrist, and/or physical therapist to refer the child back to the orthopedic surgeon, even if they are not yet due for their routine orthopedic follow-up.

When surgery is needed, appropriate patient selection and assessment are crucial to avoid recurrence and overcorrection. The following should be kept in mind when considering surgical intervention for varus feet in children with CP:

- If possible, surgery should be delayed until after age 8 years;
- Careful foot assessment should be performed, including flexibility and range of motion, X-rays, and CGA if possible (or observational gait assessment if not);
- Correction of underlying muscle imbalance is important, including appropriate muscle selection and surgical techniques;
- Bone surgery (hindfoot and/or midfoot osteotomies) should be included if there is a fixed component to the deformity.

## 6. Future Directions

Studies on surgical outcome of varus feet reported in the literature are difficult to interpret as a whole, partly due to inconsistent outcome measures used or poor documentation of how an outcome was measured [54]. It is highly recommended that surgical outcomes be assessed in a standardized way and that this should include a measure of function. Standardized assessments exist which are quick and easy to perform in a clinical setting, such as the FPI-6 (standing foot position) [30,31], the EVGS (observational gait tool) [28], and the Functional Mobility Scale (CP-specific measure of ambulatory function for home, school/work, and community distances, administered verbally) [58,59].

With continued improvements in the ability of CGA to measure various components of foot position and motion in the coming years, pre-operative planning will likely be better customized to an individual patient’s deformity and outcomes improved. More sophisticated 3D foot models could also be used for more detailed assessment of surgical outcomes in the future. 3D GCA data are also being used to develop musculoskeletal models based on an individual patient’s anatomic morphology, joint motion during gait, and even selective motor control, in hopes of simulating various surgeries and predicting their outcome [60,61]. Such advances have the potential to greatly improve surgical outcomes in patients with CP.

## Figures and Tables

**Table 1:** Muscle contributions of the anterior and posterior tibialis muscles to varus foot posture during gait. (Data from Michlitsch, et al., 2006 [22]).

Phasic Occurrence of Dysfunction (N = 88)				
	Stance and Swing	Stance	Swing	Total
Anterior tibialis	25 (28%)	5 (6%)	0 (0%)	30 (34%)
Posterior tibialis	20 (23%)	3 (3%)	6 (7%)	29 (33%)
Both	23 (26%)	0 (0%)	4 (7%)	27 (31%)
Neither	2 (2%)	0 (0%)	0 (0%)	2 (2%)

## Data Availability

No new data were created or analyzed in this study. Data sharing is not applicable to this article.

## Abbreviations

The following abbreviations are used in this manuscript:

CP	Cerebral palsy
CPUP	Cerebral Palsy Follow-up Program
GMFCS	Gross Motor Function Classification System
CGA	Computerized gait analysis
AP	Anterior posterior
SEMLS	Single event multilevel surgery
SPOTT	Split posterior tibialis tendon transfer
PTTL	Posterior tibialis tendon lengthening
SPLATT	Split anterior tibialis tendon transfer
EVGS	Edinburgh Visual Gait Scale
FPI	Foot Posture Index
